# Interactions between rodents and weeds in a lowland rice agro‐ecosystem: the need for an integrated approach to management

**DOI:** 10.1111/1749-4877.12395

**Published:** 2019-07-29

**Authors:** Nyo Me HTWE, Grant R. SINGLETON, David E. JOHNSON

**Affiliations:** ^1^ Plant Protection Division, Department of Agriculture Ministry of Agriculture, Livestock and Irrigation Myanmar; ^2^ International Rice Research Institute DAPO Box 7777 Metro Manila Philippines; ^3^ Natural Resources Institute University of Greenwich, Chatham Maritime Kent UK

**Keywords:** *Bandicota bengalensis*, Myanmar, rice, rodents, weeds

## Abstract

Rodents and weeds are important pests to rice crops in Southeast Asia. The interaction between these 2 major pests is poorly documented. In temperate cereal systems, seeds of grass weeds can be an important food source for rodents and weed cover along crop margins provides important refuge for rodents. In 2012 and 2013, a replicated study (*n* = 4) in Bago, Myanmar compared 4 treatments (rodents and weeds; no rodents and weeds; rodents and no weeds; no rodents and no weeds) each of 0.25 ha in transplanted rice. Weeds were managed with hand weeding in the wet season, and hand weeding and herbicides in the dry season. Plastic fences were installed to exclude rodents. We examined the weed cover and relative abundance of weed species, rodent damage, rodent population dynamics and rice yield loss caused by rodents and weeds. The dominant rodent species was *Bandicota bengalensis*. In the dry season, *Cyperus difformis* was dominant at the tillering stage and *Echinochloa crus‐galli* was the dominant weed species at the booting stage. In the wet season *E. crus‐galli* was a dominant weed throughout the season. Damage by rodents was higher in the dry season. There were larger economic benefits for best weed management and effective rodent control in the dry season (258 US$/ha) than in the wet season (30 US$/ha). Concurrent control of weeds in and around rice fields combined with coordinated community trapping of rodents during the early tillering stage and ripening stage of rice are recommended management options.

### INTRODUCTION

Rice is the staple food of more than half of the global population. More than 3.5 billion people depend on rice for more than 20% of their daily calories (GRiSP 2013). Myanmar is a major rice producer and this is the most important crop in the country, where agricultural production contributes 24% to GDP (MOAI 2014). The Ayeyarwady delta, comprising the regions of Ayeyarwady, Bago and Yangon, contributes 47% of the total rice production in Myanmar (MoAI 2014). The exploitable yield gap in farmers' fields for rice in the Bago region of Myanmar has been reported as 37%, indicating that rice productivity is considerably below potential (Stuart *et al*. 2016). Pests are one of the main constraints for rice production in the Ayeyarwady delta (Brown *et al*. 2008); however, there have been few efforts to quantify the impact of pests in the delta. Elsewhere in the tropics, weeds and rodents are major concerns to farmers due to the crop losses incurred in rice production systems in Asia and Africa (Adesina *et al*. 1994; Oerke & Dehne 2004; Singleton 2003). Globally, rice yield losses due to pests have been estimated at 40%, of which weeds have the highest loss potential (Pimentel *et al*. 2001). The worldwide estimated loss in rice yield from weeds is approximately 10% of the total production (Oerke & Dehne 2004). In Southeast Asia, rodents are often listed as the most important pest of rice (John 2014), mainly because farmers are not aware of effective management practices (Singleton *et al*. 2007).

Weeds often are ranked the number one pest by farmers in the Ayeyarwady delta (Htwe *et al*., unpublished data). Common weeds of rice in Myanmar include the grasses *Echinochloa crus-galli*, *Echinochloa colona*, *Ischaemum rugosum* and *Leptochloa chinensis*, the sedges *Cyperus difformis*, *Cyperus iria* and *Fimbristylis miliacea*, and the broadleaved weeds *Alternanthera* spp., *Ludwigia* spp. and *Eclipta prostrata* (Rao *et al*. 2007). The damage and yield loss in rice caused by weeds is poorly documented and farmers often have limited options for effective weed management other than manual weeding, combined with the cultural methods of land preparation and flooding the soil. Since 2012, herbicides have become more readily available in Myanmar and farmers in the delta have begun to use them in rice, primarily in the dry season crop often together with direct seeding rather than transplanting. Farmers, however, have limited knowledge about how to select the appropriate herbicide and of how to apply them safely and efficiently.

Rodents are major pests of rice in the delta area, and often are ranked second behind weeds (Brown *et al*. 2008). *Bandicota bengalensis* (Gray, 1835) is the dominant rodent pest in the lowland rice-based agro-ecosystem in Myanmar (Htwe *et al*. 2013). Farmers mainly rely on using zinc phosphide poison and rat hunters for controlling rodent populations; however, farmers' actions are primarily reactive rather than proactive because they act only when rodent numbers are high (Htwe *et al*. 2013). Htwe *et al*. (2013) reported on a major outbreak of rodent populations in the lower delta of Myanmar that occurred in 2009 and 2010. The outbreak was associated with asynchronous planting of rice crops interspersed with large tracts of cropland abandoned because of the high human casualty rate caused by cyclone Nargis in 2008. The authors suggested that weeds in these abandoned fields could have provided a significant refuge area and alternative food source (grass seeds) for rodents. There were no quantitative data, however, on the contribution of weed seeds to rodent population growth.

The current study explores the interaction between rodents and weeds in lowland irrigated rice fields in the Ayeyarwady delta, Myanmar. A balanced, replicated, experimental study was conducted with 4 treatments: no control of rodents or weeds; rodents only are controlled; weeds only are controlled; and both rodents and weeds are controlled. We hypothesize that, first, weed infestations in and around rice crops provide refuge areas for rodent pests and a source of high protein seeds that may benefit the breeding of rodent populations. We therefore predict that areas with weed infestations will have greater rodent densities and a larger proportion of breeding females than sites where weeds are controlled. Second, we hypothesize that high rodent densities result in many cut rice tillers. This opens the crop canopy and benefits weeds due to reduced competition. We therefore predict that crops with greater rodent damage will also have higher weed densities than at sites where rodents are controlled.

### MATERIALS AND METHODS

#### Study site

The study was conducted in Pyay Township in Bago region, Myanmar. In the area, the main cropping pattern is 2 rice crops a year: 1 in the wet season (monsoon) and 1 in the dry season. Crops are commonly established in both seasons by transplanting of rice seedlings from a nursery. Pyay Township was selected because the Department of Agriculture staff reported that rice crops in the area have a history of weed and rodent problems. We met with the farmers' community in Zee Oat Village (18°51′16.6″N, 95°16′30.1″E) and selected farmers who were willingly to collaborate with us. The size and shape of their farms varied. The mean farm size was approximately 8 ha.

### Methods and design

*Study design and replication*

We imposed weed management (best weed management [BWM] and farmer weed management [FWM]) as the main factor and rodent management (either no rodents present because they were excluded from a rice field by using a plastic fence, or rodents present [no rodent management]) as a split factor in the wet and the dry season. All crop management practices were similar. We imposed the following treatment for rodents at BWM and FWM sites: (i) rodents were excluded in the BWM plots using a plastic fence (BWM + No rodents); (ii) no rodent management in the BWM plots (BWM + Rodents); (iii) rodents were excluded in the FWM by using a plastic fence (Fig. 1) (FWM + No rodents); and (iv) FWM plots where rodents were not excluded (FWM + Rodents). A replicated study ($n$ = 4) compared the 4 treatments. The 16 plots were each 0.25 ha (Fig. 1).

*Weed management*

The best weed management (BWM) plots in the wet season were weeded 3 times by hand (15, 45 and 90 days after transplanting). The third weeding at 90 days after transplanting was required due to the grass weed *E. crus-galli*, which was abundant.

In the dry season, plots were weeded 3 times by hand (15, 45 and 90 days after transplanting), and the herbicide bispyribac-sodium (Nominee @ 25 g ai/ha) was applied to the rice field 25 days after transplanting. The earth bunds surrounding BWM plots were treated with glyphosate 7 days before transplanting to kill weeds. In the BWM plots, border areas were also weeded to reduce the dilution effect of treatment from other neighboring fields (Fig. 1).

The FWM plots were hand weeded twice (30 and 90 days after transplanting) in the wet season and 3 times (15, 45 and 90 days after transplanting) in the dry season.

*Rodent exclusion*

A plastic fence was installed around the perimeter of the 0.25-ha rat exclusion plots 10 days after transplanting rice in the BWM ($n$ = 4) and FWM ($n$ = 4) plots. The fence was made from polyethylene, was 650 mm high and was supported by bamboo poles and string. The bottom of the fence was buried in the soil to a depth of 100 mm (Fig. 2).

**Figure 1** The study design

In the rodent exclusion plots, 20 plastic kill-traps (Kness USA, Big-Snap E Rat Trap) were set for 3 consecutive nights to remove the rodents inside the exclusion plot before the plastic fence was installed.

No control of rodents was undertaken during the cropping season; however, hunters trapped rodents occasionally at night and the numbers of rodents caught by them were recorded in the BWM and FWM plots.

*Weed species composition and cover*

Five sampling points were randomly selected within each 0.25-ha plot. A visual scoring of percentage ground cover of weeds present above and below the crop canopy was taken from a (1 m × 1 m) quadrat at tillering and booting stages and 2 weeks before the harvest of the rice (ripening), and the percentage ground cover of the different weed species was recorded (Elazegui *et al*. 1990). Weed identification was mainly based on Caton *et al*. (2010). Percentage ground cover of rice present in quadrants was visually estimated. The weed species composition (as grass, sedge and broadleaf) and rice was expressed as a percentage of the total ground area at each sampling point.

*Estimating rodent population index and abundance*

A population index of rodents was estimated using tracking tiles and counts of active burrows. A tracking tile consisting of a bamboo mat (0.3 × 0.3 m) was smeared with mud (Fig. 3) and set at 10-m intervals. The tiles were set for 3 consecutive nights at the tillering, booting and ripening stages of the rice crop. Two lines of 200 m were set in each block: 1 along the bund of a secondary irrigation channel and 1 along a tertiary channel. The number of rodent tracks was recorded once a day. The tile with the presence of tracks (or) feces (or) both was noted as “1” (presence) and no tracks (or) feces was identified as “0” (absence).

The number of active burrows in each plot was counted at the tillering, booting and ripening stages of the rice and 1 week after harvest. Each opening was marked with a numbered bamboo stick then plugged with a thin layer of mud on the first night. The number of freshly re-opened entrances was recorded the following day. Two lines of 100 m each were monitored: 1 along a bank of a secondary irrigation channel and 1 along a tertiary channel.

Kill-trapping was done 2 weeks after harvest to identify the rodent species and their relative abundance in the BWM and FWM treatments (for details see Htwe *et al*. 2012). The breeding condition of female rodents was assessed at necropsy by recording the number of placental scars, the number of embryos and the trimester of the embryos.

*Rodent damage assessment*

Damage to rice caused by rodents was assessed at tillering, booting and ripening crop stages by stratified random sampling. Four transects began at one end of the rice crop and continued through to the middle of the field. The beginning of the transects were at 1 m, 14 m, 27 m and 40 m spacing along the edge of the crop. Damage to plants was assessed at 3 strata: 5 m, 15 m and 25 m into the crop. Ten rice “hills” were assessed

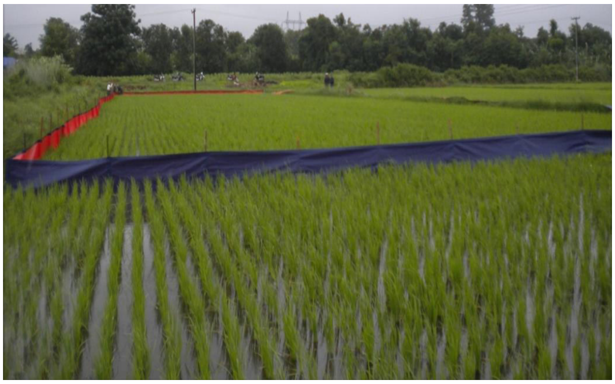

**Figure 2** A plastic fence used to provide a rodent exclusion plot

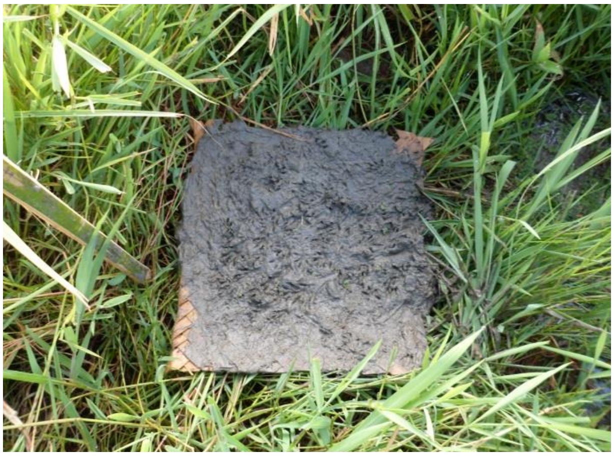

**Figure 3** A tracking tile consisting of a bamboo mat (0.3 × 0.3 m) was smeared with mud at each stratum. The hills were selected at 1-m intervals, and the spacing between hills was noted to standardize the minimum number of total hills (20 hills) in 10 sampling points. The number of damaged tillers (rice stems), regenerated tillers and undamaged tillers were recorded at each sampling point.

*Rice grain yield assessment*

The grain yield of rice in each treatment was estimated from 5 crop-cuts of 10 $m^2$ (2.5 m × 4 m) randomly selected in each plot just prior to harvest. After threshing, winnowing and drying, the grain was weighed and expressed in t/ha at 14% moisture.

*Cost and benefit analysis of rodent and weed management*

Cost of inputs (fertilizer and pesticides), labor cost, rice yield and selling price of rice were recorded for both BWM and FWM plots. Yield differences among different treatments were calculated. The costs and benefits (total cost of weed management deducted from the market value of harvested rice yield loss) of (i) BWM+ no rodents, (ii) BWM + rodents, (iii) FWM + No rodents and (iv) FWM + rodents were compared among treatments to estimate the benefit of weed and rodent management, respectively.

### Data analyses

Weed species composition was based on the assessment of % ground cover of the different weed species at sampling times. The weed management (BWM and FWM), season (wet and dry) and crop stages (tillering, booting and ripening stage) were used as predictors to test their effect on the following parameters: weed cover and percentage of rodent damage to the crop. Cohen's *d* formula was used to estimate the effect size. A general linear model was used to analyze the effect of weed management and rodent management on yield. Two-way analysis of variance was used to determine the effect of season and treatment on rodent abundant (the number of active burrows and data from tracking tiles). The $\chi^2$-test was used to examine differences in occurrence of breeding female rodents (lactating and/or pregnant as determined at necropsy) between BWM and FWM plots. Linear regression analyses were conducted to determine whether the rodent damage could also be related to the cover of weeds in different treatment combinations. All statistical analyses were carried out using SPSS version 15.0.

## RESULTS

### Weed species composition and cover

Weed growth varied according to season and weed management treatment. In the wet season, the grass *E. crus-galli* had the greatest ground cover, with 24%, 16% and 17% cover, respectively, at the 3 different crop stages, followed by the fern *Marsilea minuta*, with 13%, 15% and 10% cover, respectively, and the sedge *C. difformis*, with 9%, 11% and 6% cover, respectively (Table 1). In the dry season, at the tillering stage the sedge *C. difformis* (31% cover) had the greatest ground cover, while at the booting stage it was the grass, *E. crus-galli* (24% cover), and at ripening it was the broad leaf *Limnocharis flava* (26% cover).

The mean total weed cover (%) in the BWM plot was greater ($F_{1,15}$ = 4.163; $P$ = 0.037) in the wet season than the dry season (Fig. 4). The total weed cover (%) in FWM and BWM was not significantly different in the wet season (the effect size, Cohen *d* value was 0.26); however, in the dry season the total weed cover was greater in FWM than BWM ($F_{1,7}$ = 8.49; $P$ = 0.03; effect size Cohen *d* value = 1.22).

At the rice tillering stage in the wet season, grasses and broadleaved species were present in FWM and BWM plots. By the booting stage, grasses and sedges were dominant in FWM, while in BWM, only broadleaves were recorded (Fig 5a). At rice ripening, grasses were recorded in FWM and BWM, while broadleaves were only recorded in FWM. In the dry season, grasses were present in FWM at all 3 crop stages, but only at the booting stage in BWM; sedges were recorded in FWM at both the tillering and booting stages (Fig. 5 (ii)).

### Rodent population index and the breeding performance of female rodents

*Rodent population index*

The tracking tiles indicated no significant difference in rodent activity between BWM and FWM in the wet season (effect size Cohen *d* value = 0.47); however, there was significantly more activity in the FWM field than the BWM field in the dry season ($F_{1,7}$ = 47.55, $P$ = 0.025; effect size= 0.819). Similar findings were observed in burrow counting methods in the wet season (the effect size was 0.057) and the dry season ($F_{1,7}$ = 4.196, $P$ = 0.038; effect size Cohen *d* value = 0.68).

*Breeding performance of female rodents*

The occurrence of breeding female rodents in the wet

**Table 1** Weed species composition (as % ground cover) in farmer weed management plots at different rice growth stages (tillering, booting and ripening) during the 2012 wet and 2013 dry seasons in Pyay Township, Myanmar

| Species | Mean ground cover (%) | | |
|---|---|---|---|
| | Tillering | Booting | Ripening |
| 2012 Wet Season | | | |
| *Chrysopogon aciculatus* | 0.0 | 5.3 | 13.8 |
| *Cyperus difformis* | 9.0 | 10.7 | 6.4 |
| *Cyperus iria* | 1.5 | 6.7 | 7.4 |
| *Echinochloa colona* | 0.0 | 5.3 | 2.1 |
| *Echinochloa crus-galli* | 23.9 | 16.0 | 17.0 |
| *Eichhornia crassipes* | 3.0 | 0.0 | 0.0 |
| *Fimbristylis miliacea* | 1.5 | 0.0 | 0.0 |
| *Hymenachne amplexicaulis* | 1.5 | 0.0 | 0.0 |
| *Ipomoea aquatic* | 6.0 | 2.7 | 6.4 |
| *Isachne globose* | 9.0 | 5.3 | 0.0 |
| *Leersia hexandra Sw.* | 0.0 | 1.3 | 9.6 |
| *Limnocharis flava* | 1.5 | 13.3 | 11.7 |
| *Ludwigia adscendens* | 7.5 | 9.3 | 7.4 |
| *Ludwigia hyssopifolia* | 1.5 | 0.0 | 0.0 |
| *Marsilea minuta* | 13.4 | 14.7 | 9.6 |
| *Monochoria vaginalis* | 13.4 | 0.0 | 0.0 |
| *Scirpus grossus* | 3.0 | 5.3 | 4.3 |
| *Scirpus juncoiedes* | 4.5 | 0.0 | 0.0 |
| *Sphenoclea zeylanica* | 0.0 | 4.0 | 4.3 |
| 2013 Dry season | | | |
| *Chrysopogon aciculatus* | 1.1 | 1.1 | 2.5 |
| *Cyperus difformis* | 27.3 | 13.5 | 3.7 |
| *Cyperus iria* | 0.0 | 2.8 | 3.7 |
| *Echinochloa colona* | 0.0 | 0.0 | 3.1 |
| *Echinochloa crus-galli* | 9.1 | 19.1 | 8.1 |
| *Fimbristylis miliacea* | 0.0 | 3.9 | 4.4 |
| *Hymenachne amplexicaulis* | 0.0 | 0.0 | 1.9 |
| *Ipomoea aquatic* | 0.0 | 3.4 | 4.4 |
| *Isachne globose* | 0.0 | 0.0 | 1.9 |
| *Limnocharis flava* | 27.3 | 17.4 | 29.8 |
| *Ludwigia adscendens* | 6.8 | 5.6 | 6.2 |
| *Ludwigia hyssopifolia* | 0.0 | 0.0 | 9.3 |
| *Marsilea minuta* | 7.9 | 11.2 | 6.2 |
| *Monochoria vaginalis* | 4.6 | 0.0 | 0.0 |
| *Scirpus juncoiedes* | 9.1 | 7.3 | 6.2 |
| *Sphenoclea zeylanica* | 3.4 | 5.1 | 8.1 |
| Unknown spp. (broad leaf species) | 3.4 | 9.6 | 0.6 |

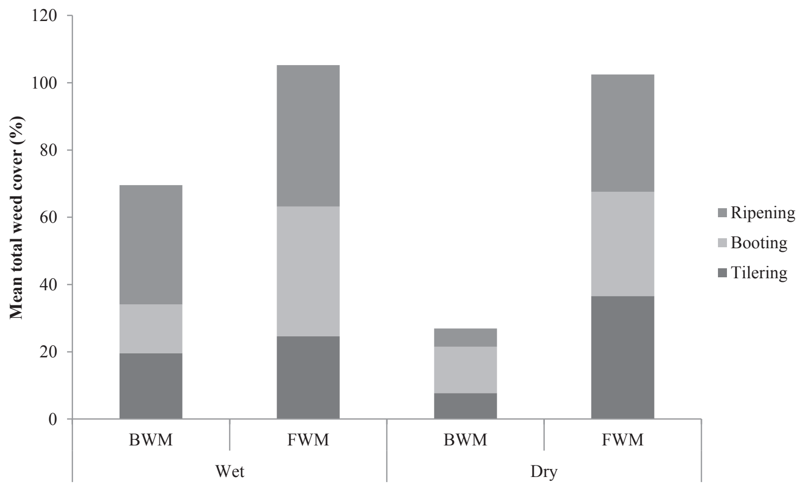

**Figure 4** Mean weed cover in best weed management (BWM) and farmer weed management (FWM) plots in the wet and the dry season at 3 growth stages of the rice crop

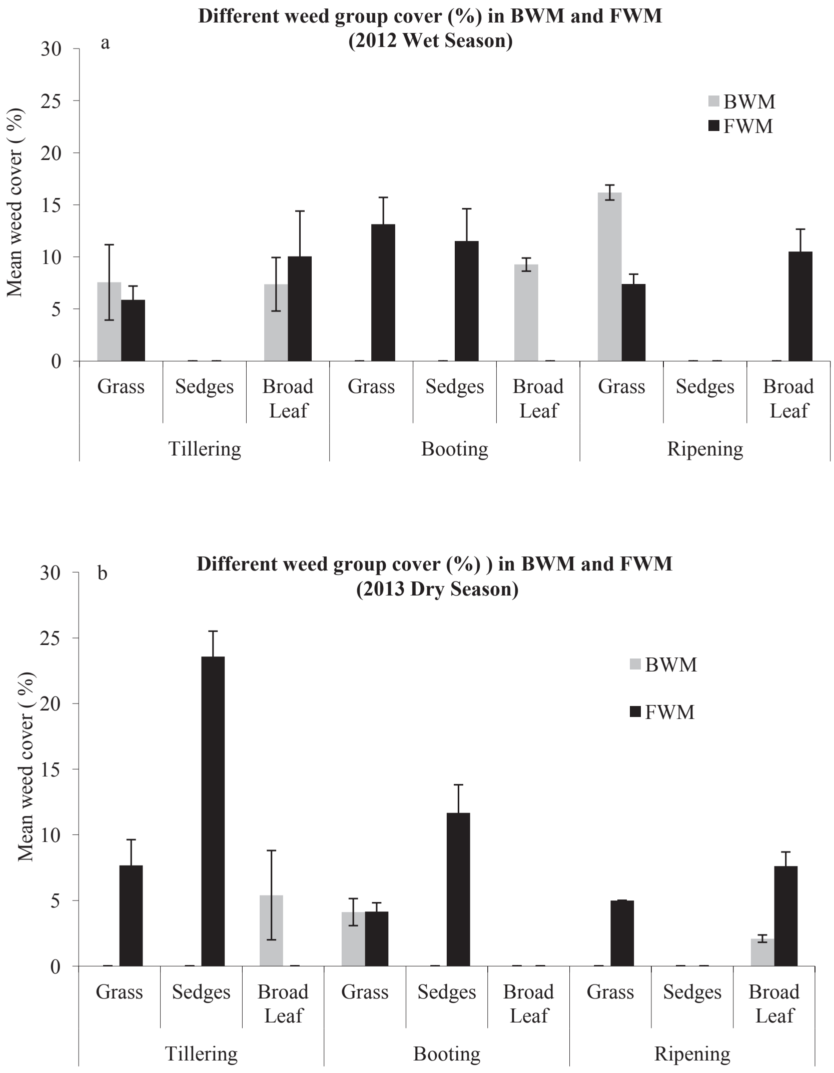

**Figure 5** Mean cover of different groups of weeds at 3 growth stages of rice for: (a) 2012 wet season and (b) 2013 dry season, in Pyay Township, Myanmar with SE bars season did not differ between BWM and FWM; however, in the dry season the occurrence of breeding females was greater in FWM than in BWM ($P < 0.001$; Table 2). The mean time of conception of rodents was at the ripening stage in both the BWM and FWM plots. The data on rodent reproduction shows greater numbers of breeding females in FWM plots than the BWM plots, and the major time of conception being the ripening stage of rice.

### Rodent damage to the rice crop

There was significantly ($F_{1,15}$= 4.163; $P$ =0.037; effect size Cohen's $d$ value = 0.92) less rodent damage (%) in the wet (8.05 ± 2.76) than the dry season (15.4 ± 2.83; Fig. 6). In the wet season, rodent damage did not differ significantly (effect size Cohen's $d$ value= 0.15) between crop stages, with the greatest damage at the tillering stage (4.14 ± 1.9).

### Relationship between rodent damage and weed cover

There was a weak positive association between rodent damage (%) and the weed cover in both the wet ($F_{1,24}$ = 7.64; $P$ = 0.042; $R^2$ = 0.133) and dry seasons ($F_{1,24}$ = 5.43; $P$ = 0.029; $R^2$ = 0.198).

### Yield loss by weeds and rodents

In the wet season, the yield difference among BWM + No rodents and FWM + Rodents plots differed significantly. In the dry season, the comparison among different treatments indicated that the yield from BWM + No rodents was significantly greater than from other plots. However, the yield between FWM + No rodents and FWM + Rodents was not significantly different (Table 3).

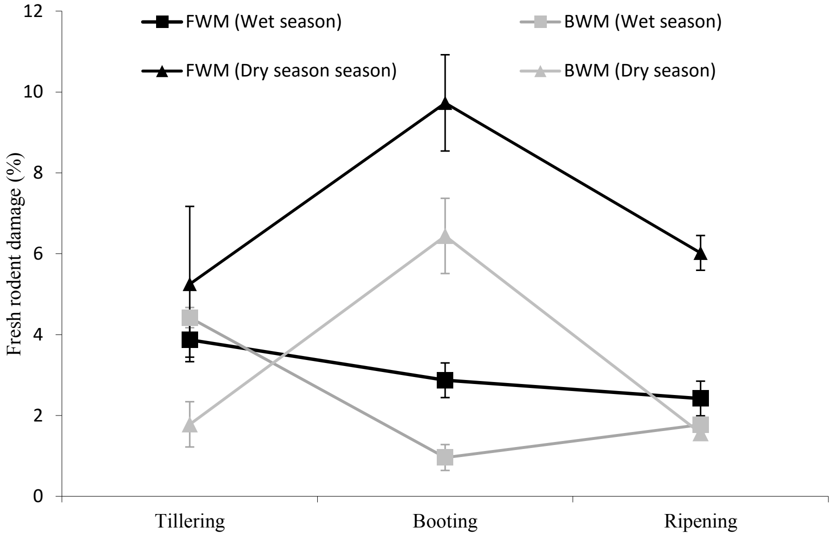

**Figure 6** Rodent damage (%) to the growing rice crop at 3 growth stages of the rice crop in best weed management (BWM) and farmer weed management (FWM) plots in the 2012 wet and 2013 dry season in Pyay Township, Myanmar with SE bars

**Table 2** Breeding performance of female *Bandicota bengalensis* in best weed management (BWM) plots and farmer weed management (FWM) plots

| Crop season | Treatment | Number of rodents trapped | Proportion of females (%) | Number of adult females | Number of pregnant females | Number of lactating females | Conception time (crop stage) |
|---|---|---|---|---|---|---|---|
| 2012 (Wet) | BWM | 12 | 25 ($n$ = 3) | 3 | 0 | 1 | Ripening |
| 2012 (Wet) | FWM | 23 | 44 ($n$ = 10) | 10 | 0 | 3 | Ripening |
| 2013 (Dry) | BWM | 40 | 33 ($n$ = 10) | 5 | 2 | 1 | Ripening |
| 2013 (Dry) | FWM | 54 | 46 ($n$ = 25) | 14 | 7 | 3 | Ripening |

The conception time was calculated by backdating based on whether a pregnant female was at 1st, 2nd or 3rd trimester.

#### Cost and benefit of rodent and weed management

The cost–benefit from excluding rodents and managing weeds in the dry season was greater than in the wet season ($F_{1,18}$ = 44.803; $P$ < 0.001), with an estimated benefit of 198.07 ± 18.43 US$/ha compared to 282.25 ± 37.22 US$/ha in the dry season.

In the wet season, the benefit of weed and rodent management did not differ significantly among all treatments. The difference of the benefit between BWM + No rodents was significantly higher by 50.65 US$/ha than FWM + Rodents; however, FWM + Rodents provided more benefit (104.89 US$/ha) than BWM+ Rodents in the wet season (Table 3).

In the dry season, the benefit of weed and rodent management differed significantly among all treatments ($F_{1,15}$ = 9.196; $P$ = 045). The largest difference was the comparison of BWM + No rodents and FWM + Rodents (250.62 US$/ha). The difference of the benefit between BWM + Rodents and FWM + Rodents was 113.28 US$/ha, while the difference between FWM + No rodents and FWM + Rodents was 42.37 US$/ha (Table 3).

### DISCUSSION

#### Need for concurrent management of rodents and weeds

The highest rodent infestations occurred in rice fields where the weed cover was greatest in the dry season. The greater weed cover in the dry season than in the wet season was associated with increased rodent damage and yield loss. It was notable that grasses were present throughout the crop in the FWM plots in the dry season and this coincided with a higher incidence of breeding female rodents in these plots. In the wet season, the presence of rodents was the main factor influencing negative cost–benefits. Cost and benefit analysis suggested that rodent management could be more efficient if it was combined with BWM in the dry season but less so in the wet season when weed biomass was lower and FWM

**Table 3** The difference in mean yields between treatments and the relative cost–benefit achieved by managing weeds and rodents in rice crops in Pyay Township, Myanmar

| Season | (1) Treatment | (2) Treatment | Significance | (3) Yield mean difference (1–2) (t/ha) | (4) Price of (US$/t) | (5) Price of weed management (US$/ha) | Benefit (3 * 4) − (5) | Significance |
|---|---|---|---|---|---|---|---|---|
| Wet | BWM + No rodents | BWM + Rodents | 0.115 | 0.521 | 152.6 | 50 | 29.50 | 0.929 |
| | | FWM + No rodents | 0.841 | 0.063 | 152.6 | 35 | −25.39 | 0.771 |
| | | FWM + Rodents | 0.093 | 0.561 | 152.6 | 35 | 50.61 | 0.004 |
| | BWM + rodents | FWM + No rodents | 0.162 | −0.458 | 152.6 | 35 | −104.89 | 0.004 |
| | | FWM + rodents | 0.898 | 0.04 | 152.6 | 35 | −28.89 | 0.093 |
| | FWM + No rodents | FWM + Rodent | 0.131 | 0.498 | 152.6 | 35 | 40.99 | 0.327 |
| Dry | BWM + No rodents | BWM + Rodents | 0.054 | 0.95 | 152.6 | 70 | 74.97 | 0.027 |
| | | FWM + No rodents | 0.001 | 2 | 152.6 | 50 | 250.62 | 0.017 |
| | | FWM + Rodents | 0.001 | 2.02* | 152.6 | 50 | 258.25 | 0.021 |
| | BWM + rodents | FWM + No rodents | 0.041 | 1.02 | 152.6 | 50 | 105.65 | 0.020 |
| | | FWM + Rodents | 0.033 | 1.07 | 152.6 | 50 | 113.28 | 0.017 |
| | FWM + No rodents | FWM + Rodents | 0.912 | 0.05 | 152.6 | 50 | −42.37 | 0.112 |

The benefit–cost was based on a farm gate price of 152.6 US$/t of rice. BWM + No rodents = Best weed management with rodents excluded by installing a plastic fence. BWM + Rodents = Best weed management in an open field; rodents were not excluded by installing plastic fence. FWM + No rodents = Farmer weed management with rodents excluded by installing a plastic fence. FWM + Rodents = Farmer weed management in an open field; rodents were not excluded by installing a plastic fence.

was just as effective. Our results are, therefore, consistent with the hypothesis that there is a positive relationship between high weed infestation and high rodent damage. These findings have important implications for strategies to reduce the rice yield losses by managing weeds and rodents in rice concurrently, especially in the dry season.

To our knowledge, this is the first time that an experimental study at a biological meaningful scale has demonstrated the additive negative effects of rodent and weed interactions in rice fields of farmers, be it mainly in the dry season. There has been one previous report of rodents causing high damage to rice in control plots designed to measure weed losses in the Philippines but rodent–weed interactions was not the focus of the original experimental design (Drost & Moody 1982). The need for integrated pest management across a spectrum of pests has been recommended for insects and weeds (e.g. Khan *et al*. 2011), and weeds and rodents are an important consideration when managing margins of rice crops to grow plants that attract beneficial predators of insects, particularly spiders (Horgan *et al*. 2016). Furthermore, there has been research on weed–bird interactions in lowland rice that indicates that good weed management will reduce crop losses by granivorous birds (Rodenburg *et al*. 2014). Reduced plant cover on bunds of rice crops and fence lines of wheat crops is part of a recommendation for ecological management of rodents (Brown *et al*. 2017) because rodent pests of cereal crops show a clear habitat preference for crop margins with high weed biomass (Ylönen 2002; Jones *et al*. 2017).

Studies in temperate cereal cropping systems suggest that seeds of grass weeds are an important food source for rodents when cereal seeds are in short supply and the seeds could be a trigger for breeding (Bomford 1987a; Tann *et al*. 1991). In our study, grass weeds were more abundant in FWM plots than BWM plots in the dry season. Little is known, however, about the importance of weeds on the population dynamics of rodent pests in rice-based cropping systems in Southeast Asia. Htwe *et al*. (2014) reported rice and grasses to be the main components of the diet of *Rattus tanezumi* (Temiminck, 1844) in lowland rice crops in the Philippines. Reports from weed studies have mentioned an apparent association between high weed infestations and high rodent damage to rice crops (Drost & Moody 1982; Heinrichs *et al*. 1995), but quantitative data are lacking. Rodent damage to rice crops is often patchy, with areas of high losses frequently occurring towards the middle of the rice crop (Fall 1977; Miller *et al.* 2008; Buckle 2015; Jones *et al*. 2017). We have observed that where rodent damage is high, the rice crop leaf canopy has been "opened up" and weeds, which then are able to grow rapidly due to reduced competition for light, become a major problem for the remaining rice crop. In Africa and Asia, weed competition in the earlier stages of rice growth results in higher yield losses than later competition from weeds (Johnson *et al*. 2004; Chauhan & Johnson 2011).

In this study, the FWM involved 3 manual weeding activities in the dry season, which reflected the prevailing local practices, and yet, despite these investments, the dry season yields could be increased by more than twice through the additional use of a herbicide in BWM. This supports the case Rao *et al*. (2007) made that weed management in rice crops needs to be recognized as a high priority if major losses are to be avoided. Rice crop losses due to rodents are less well defined. In one study in Myanmar, more than 47% of farmers reported that among the pests of rice, it was rodents that caused the most damage (Brown *et al*. 2008) and an episodic but extended rodent outbreak in the Ayeyarwady delta led to many farmers suffering losses of 25–45% to their rice crops over 2 seasons (Htwe *et al*. 2013). Greater rodent losses including complete loss of rice and other crops have also been reported in upland, northern areas of Myanmar following a major bamboo flowering event (Htwe *et al*. 2010), but the current study was in a markedly different agro-ecosystem. Our results, however, serve to highlight the importance of effective crop protection practices targeting rodents and weeds if farmers are to achieve greater yields in the lowland areas. Brown *et al*. (2004) conducted a manipulation study in southern Australia that reduced weed and grass biomass around the perimeters of irrigated rice fields on the assumption that such vegetation provides nesting sites and alternative food for house mice (*Mus domesticus* Rutty, 1772). They reported a benefit–cost of 9:1 for rice production in terms of profitability. In our study, rodent damage and yield loss was substantially lower only in the dry season in the plots of BWM compared to the farmer practice for weed management, leading to higher returns of 258 US$/ha.

In terms of cost and benefit, FWM practices in the wet season could be more economical than the 3 "hand weedings" in BWM plots if combined with the rodent management. The average yield, however, during the wet season (3.1 ± 0.10 t/ha) in our study was lower than the optimum yield of 5 t/ha reported by Stuart *et al*. (2016). Other crop management practices, such as crop nutrition, may also need to be improved to achieve such yield levels.

Our findings suggest that FWM practices in the dry season were not sufficient to control weeds and this subsequently was associated with greater rodent damage (farmers lost on average 258 US$/ha in FWM plots). Farmers would still lose approximately 236 US$/ha if they managed weeds by best practices but did not implement management actions for rodents. We recommend that in conjunction with BWM, rodent management practices such as digging burrows and community trapping should be conducted at the land preparation time and the early tillering stage before the breeding season of the main target species, *B. bengalensis*.

Our study was a researcher-managed trial in the fields of farmers. An adaptive research (AR) management approach has been introduced in Myanmar through which researchers and farmers learn and work together in a participatory approach to adopt and adapt new technologies (see detail in Flor *et al*. 2017). An interesting future study would be to test large-scale weed and rodent management demonstration plots led by farmers using an AR approach with the assistance of researchers and extension specialists. This could then be followed with the development of an adoption pathway for broad scale and economically efficient integrated rodent and weed management approaches.

### Effects of weed management on breeding and spatial behavior of rodents

Occurrence of breeding females was higher in FWM plots than in BWM plots, and the major time of conception was the ripening stage of rice. Previous studies of the diet and breeding performance of *M. domesticus* (Bomford 1987a,b), *R. tanezumi* and *Rattus argentiventer* (Robinson & Kloss, 1916) (Htwe *et al*. 2012) in rice crops report that weed seeds are likely to be an important source of high quality food that either triggers or promotes their breeding. Our findings support these findings; the presence of grass weeds increased the occurrence of breeding female *B. bengalensis* in FWM plots in the dry season. This might be tested through providing shelter for nesting sites and/or a source of high-quality food through their seed production. Further research is required on the influence of diet quality in rice landscapes on the reproductive performance of *B. bengalensis*. Certain weed species may encourage rodents to breed and, for example, the sedge *C. difformis* is able to produce seeds after approximately 30–35 days after germination and could provide an early food source.

The tracking tile and burrow count results did not provide a clear picture of the abundance of rodents in BWM and FWM plots in the wet season. The spatial behavior of *B. bengalensis* may be influenced by their perception of predation risk. Higher weed cover may simply encourage rodents to seek harbor there because of a perceived reduced risk of predation (Jones *et al*. 2017; Krijger *et al*. 2017). Again, little is known about how the behavior of *B. bengalensis* at a local scale is affected by a landscape of fear, so further research is warranted.

In the Mekong Delta of Vietnam, My Phung *et al*. (2010) found that damage to rice tiller by the rice-field rat (*R. argentiventer*), can reduce the yield at any crop stage depending on the severity of rodent damage. The relative yield loss caused by rodents in our study was greater than in the Mekong study. The mean body weight of *B. bengalensis* is up to 400 g, approximately double that of *R. argentiventer*, plus the rodent cuts and hoards the rice panicles at the ripening and harvesting stages (Sheikher *et al*. 1991; Htwe *et al*. 2017). *R. argentiventer* does not horde rice in its burrows.

### Dominant weed species

*Echinochloa crus-galli* was a common grass weed species during the wet season in our study and was also the dominant species at the booting stage in the dry season. It is a notorious grass weed of lowland rice crops due to its ability to grow rapidly. *E. crus-galli* is a grass with the C4 photosynthetic pathway and it is able to grow more rapidly than rice, which is a C3 plant, and crop yield losses due to weed competition can range from 30% to 100% in rice (Johnson *et al*. 1998). The weed composition in FWM plots shifted between the different crop stages in the dry season. Farmers are particularly aware of losses caused by *E. crus-galli* and, in consequence, they paid more attention to controlling this species. The changes in the weed composition in the dry season may have been due to farmers preferentially removing this dominant species, but also the composition of weed growth may have been affected by flooding regimes and temperature.

The sedge weed *C. difformis* was the most common weed at the tillering stage in the dry season. This sedge and the grass *E. crus-galli* are considered among the most serious weeds of rice in the world (Holm *et al*. 1991). The broad leaf weed *L. flava* tends to be less of a problem in rice crops as it is comparatively easy to control. Both *E. crus-gali* and *C. difformis* need to be managed carefully as they grow rapidly and are very competitive with the crop for nutrients and light. Increasingly, farmers are facing higher labor costs for hand weeding, and are becoming more reliant on the use of herbicides. However, farmers have limited knowledge about herbicide application; increased awareness of which herbicide to use at what time is urgently needed.

### CONCLUSION

Our findings show a clear interaction between rodents and weeds in a lowland irrigated rice agro-ecosystem. We also demonstrated greater crop losses from the interaction of rodents and weeds in the dry cropping season than in the wet season. In the dry season when rodent densities and weed biomass were highest, rodent abundance was highly associated with high weed infestation, and the economic returns were substantially higher in the BWM plots that had no rodents. In plots where weeds were managed following farmer practice, those with rodents had considerably higher weed cover and considerably less rice yields.

A combination of increased cropping intensity and increased risk of crop pests associated with extreme climate events (see Htwe *et al*. 2012) and climate change (Rodenburg *et al*. 2011), is likely to increase crop losses caused by weeds and rodents in lowland rice. Our findings clearly highlight the need to develop and promote an integrated approach to weed and rodent management. This applies not only to Myanmar but to many of the lowland rice-growing areas in Asia where a growing human population will require a 70% increase in global food production by 2050 on current agricultural land (FAO 2009; GRiSP 2013; McKenzie & Williams 2015).

### ACKNOWLEDGMENTS

All data collection was done by the Myanmar Department of Agriculture (DoA) staff, Plant Protection Division staff and final year students from Yezin Agriculture University, Hlegu campus (2013). Without their efforts, the study could not have been completed. We thank weed and rodent researchers from the Plant Protection Division, Plant Protection staff from Pyay District, and DoA staff from Pyay Township for their support during this study. Special thanks to Dr Niamh Quinn, for sharing her experience on field studies in Indonesia and the Philippines. This research was funded by the Swiss Agency for Development and Cooperation through the CORIGAP project (Grant no. 81016734).

**Cite this article as:**

## Cite this article as:

Htwe NM, Singleton GR, Johnson DE (2019). Interactions between rodents and weeds in a lowland rice agro‐ecosystem: the need for an integrated approach to management. *Integrative Zoology* **14**, 396–409.
